# How to Conduct a Systematic Review: A Narrative Literature Review

**DOI:** 10.7759/cureus.864

**Published:** 2016-11-04

**Authors:** Nusrat Jahan, Sadiq Naveed, Muhammad Zeshan, Muhammad A Tahir

**Affiliations:** 1 Psychiatry, Mount Sinai Chicago; 2 Psychiatry, KVC Prairie Ridge Hospital; 3 Department of Psychiatry, Bronx Lebanon Hospital Icahn School of Medicine at Mount Sinai, Bronx, NY; 4 Psychiatry, Suny Upstate Medical University, Syracuse, NY

**Keywords:** systematic reviews, meta-analysis, narrative literature review, prisma checklist

## Abstract

Systematic reviews are ranked very high in research and are considered the most valid form of medical evidence. They provide a complete summary of the current literature relevant to a research question and can be of immense use to medical professionals. Our goal with this paper is to conduct a narrative review of the literature about systematic reviews and outline the essential elements of a systematic review along with the limitations of such a review.

## Introduction and background

A literature review provides an important insight into a particular scholarly topic. It compiles published research on a topic, surveys different sources of research, and critically examines these sources [1]. A literature review may be argumentative, integrative, historical, methodological, systematic, or theoretical, and these approaches may be adopted depending upon the types of analysis in a particular study [2].

Our topic of interest in this article is to understand the different steps of conducting a systematic review. Systematic reviews, according to Wright, et al., are defined as a “review of the evidence on a clearly formulated question that uses systematic and explicit methods to identify, select and critically appraise relevant primary research, and to extract and analyze data from the studies that are included in the review” [3]. A systematic review provides an unbiased assessment of these studies [4]. Such reviews emerged in the 1970s in the field of social sciences. Systematic reviews, as well as the meta-analyses of the appropriate studies, can be the best form of evidence available to clinicians [3]. The unsystematic narrative review is more likely to include only research selected by the authors, which introduces bias and, therefore, frequently lags behind and contradicts the available evidence [5].

Epidemiologist Archie Cochrane played a vital role in formulating the methodology of the systematic review [6]. Dr. Cochrane loved to study patterns of disease and how these related to the environment. In the early 1970s, he found that many decisions in health care were made without reliable, up-to-date evidence about the treatments used [6].

A systematic review may or may not include meta-analysis, depending on whether results from different studies can be combined to provide a meaningful conclusion. David Sackett defined meta-analysis as a “specific statistical strategy for assembling the results of several studies into a single estimate” [7-8].

While the systematic review has several advantages, it has several limitations which can affect the conclusion. Inadequate literature searches and heterogeneous studies can lead to false conclusions. Similarly, the quality of assessment is an important step in systematic reviews, and it can lead to adverse consequences if not done properly.

The purpose of this article is to understand the important steps involved in conducting a systematic review of all kinds of clinical studies. We conducted a narrative review of the literature about systematic reviews with a special focus on articles that discuss conducting reviews of randomized controlled trials. We discuss key guidelines and important terminologies and present the advantages and limitations of systematic reviews.

## Review

Narrative reviews are a discussion of important topics on a theoretical point of view, and they are considered an important educational tool in continuing medical education [9]. Narrative reviews take a less formal approach than systematic reviews in that narrative reviews do not require the presentation of the more rigorous aspects characteristic of a systematic review such as reporting methodology, search terms, databases used, and inclusion and exclusion criteria [9]. With this in mind, our narrative review will give a detailed explanation of the important steps of a systematic review.

### Preferred reporting items for systematic review and meta-analysis protocols (PRISMA-P) checklist

Systematic reviews are conducted based on predefined criteria and protocol. The PRISMA-P checklist, developed by Moher, et al., contains 17 items (26 including sub-items) comprising the important steps of a systematic review, including information about authors, co-authors, their mailing and email addresses, affiliations, and any new or updated version of a previous systematic review [9]. It also identifies a plan for documenting important protocol amendments, registry names, registration numbers, financial disclosures, and other support services [10]. Moher, et al. also state that methods of systematic reviews involve developing eligibility criteria and describing information sources, search strategies, study selection processes, outcomes, assessment of bias in individual studies, and data synthesis [10].

### Research question

Writing a research question is the first step in conducting a systematic review and is of paramount importance as it outlines both the need and validity of systematic reviews (Nguyen, et al., unpublished data). It also increases the efficiency of the review by limiting the time and cost of identifying and obtaining relevant literature [11]. The research question should summarize the main objective of a systematic review.

An example research question might read, “How does attention-deficit/hyperactivity disorder (ADHD) affect the academic performance of middle school children in North America?” The question focuses on the type of data, analysis, and topic to be discussed (i.e., ADHD among North American middle school students). Try to avoid research questions that are too narrow or broad—they can lead to the selection of only a few studies and the ability to generalize results to any other populations may be limited. An example of a research question that is too narrow would be, “What is the prevalence of ADHD in children and adolescents in Chicago, IL?” Alternately, if the research question is too broad, it can be difficult to reach a conclusion due to poor methodology. An example of a research question that is too broad in scope would be, “What are the effects of ADHD on the functioning of children and adolescents in North America?”

Different tools that can be used to help devise a research question, depending on the type of question, are: population, intervention, comparator, and outcomes (PICO); sample, phenomenon of interest, design, evaluation, and research type (SPIDER); setting, perspective, intervention, comparison, and evaluation (SPICE); and expectation, client group, location, impact, professionals, and service (ECLIPSE).

The PICO approach is mostly used to compare different interventions with each other. It helps to formulate a research question related to prognosis, diagnosis, and therapies [12].

Scenario: A 50-year-old white woman visited her psychiatrist with a diagnosis of major depressive disorder. She was prescribed fluoxetine, which she feels has been helpful. However, she experienced some unpleasant side effects of nausea and abdominal discomfort. She has recently been told by a friend about the use of St. John’s wort in treating depression and would like to try this in treating her current depression. (Formulating research questions, unpublished data).

In the above-mentioned scenario, the sample population is a 50-year-old female with major depressive disorder; the intervention is St. John’s wort; the comparison is fluoxetine; and the outcome would be efficacy and safety. In order to see the outcome of both efficacy and safety, we will compare the efficacy and safety of both St. John’s wort and fluoxetine in a sample population for treating depression. This scenario represents an example where we can apply the PICO approach to compare two interventions.

In contrast, the SPIDER approach is focused more on study design and samples rather than populations [13]. The SPIDER approach can be used in this research question: “What is the experience of psychiatry residents attending a transgender education?” The sample is psychiatry residents; the phenomenon of interest is transgender education; the design is a survey; the evaluation looks at the experience; and the research type is qualitative. 

The SPICE approach can be used to evaluate the outcome of a service, intervention, or project [14]. The SPICE approach applies to the following research question: “In psychiatry clinics, does the combined use of selective serotonin reuptake inhibitor (SSRI) and psychotherapy reduce depression in an outpatient clinic versus SSRI therapy alone?” The setting is the psychiatry clinic; the perspective/population is the outpatient; the intervention is combined psychotherapy and SSRI; the comparison is SSRI alone; and the evaluation is reduced depression. 

The ECLIPSE approach is useful for evaluating the outcome of a policy or service (Nguyen, et al., unpublished data). ECLIPSE can apply in the following research question: “How can a resident get access to medical records of patients admitted to inpatient from other hospitals?” The expectation is: “What are you looking to improve/change to increase access to medical records for patients admitted to inpatient?” The client group is the residents; the location is the inpatient setting; the impact would be the residents having easy access to medical records from other hospitals; and the professionals in this scenario would be those involved in improving the service experiences such as hospital administrators and IT staff.

### Inclusion and exclusion criteria

Establishing inclusion and exclusion criteria come after formulating research questions. The concept of inclusion and exclusion of data in a systematic review provides a basis on which the reviewer draws valid and reliable conclusions regarding the effect of the intervention for the disorder under consideration [11]. Inclusions and exclusion are based on preset criteria for specific systematic review. It should be done before starting the literature search in order to minimize the possibility of bias.

Eligibility criteria provide the boundaries of the systematic review [15]. Participants, interventions, and comparison of a research question provide the basis for eligibility criteria [15]. The inclusion criteria should be able to identify the studies of interest and, if the inclusion criteria are too broad or too narrow, it can lead to an ineffective screening process.

### Protocol registration

Developing and registering research protocol is another important step of conducting a systematic review. The research protocol ensures that a systematic review is carefully planned and explicitly documented before the review starts, thus promoting consistency in conduct for the review team and supporting the accountability, research integrity, and transparency of the eventually completed review [10]. PROSPERO and the Cochrane Database of Systematic Reviews are utilized for registering research protocols and research questions, and they check for prior existing duplicate protocols or research questions. PROSPERO is an international database of prospectively registered systematic reviews related to health care and social sciences (PRISMA, 2016). It is funded by the National Institute for Health Research. The Cochrane Collaboration concentrates on producing systematic reviews of interventions and diagnostic test accuracy but does not currently produce reviews on questions of prognosis or etiology [16].

A detailed and extensive search strategy is important for the systematic review since it minimizes bias in the review process [17].

Selecting and searching appropriate electronic databases is determined by the topic of interest. Important databases are: MEDLARS Online (MEDLINE), which is the online counterpart to the Medical Literature Analysis and Retrieval System (MEDLARS); Excerpta Medica Database (EMBASE); and Google Scholar. There are multiple electronic databases available based on the area of interest. Other important databases include: PsycINFO for psychology and psychiatry; Allied and Complementary Medicine Database (AMED) for complementary medicine; Manual, Alternative, and Natural Therapy Index System (MANTIS) for alternative medical literature; and Cumulative Index to Nursing and Allied Health Literature (CINAHL) for nursing and allied health [15].

Additional studies relevant for the review may be found by looking at the references of studies identified by different databases [15]. Non-indexed articles may be found by searching the content of journals, conferences proceedings, and abstracts. It will also help with letters and commentaries which may not get indexed [15]. Reviewing clinical trial registries can provide information about any ongoing trials or unpublished research [15]. A gray literature search can access unpublished papers, reports, and conference reports, and it generally covers studies that are published in an informal fashion, rather than in an indexed journal [15]. Further search can be performed by selecting important key articles and going through in-text citations [15].

### Using Boolean operators, truncation, and wildcards

Boolean operators use the relationship between different search words to help with the search strategy. These are simple words (i.e., AND, OR, and NOT) which can help with more focused and productive results (poster, Jahan, et al.: How to conduct a systematic review. APPNA 39th Summer Convention. Washington, DC. 2016). The Boolean operator AND finds articles with all the search words. The use of OR broadens the focus of the search, and it will include articles with at least one search term. The researchers can also ignore certain results from the records by using NOT in the search strategy.

An example of AND would be using “depression” AND “children” in the search strategy with the goal of studying depression in children. This search strategy will include all the articles about both depression and children. The researchers may use OR if the emphasis of the study is mood disorders or affective disorders in adolescents. In that case, the search strategy will be “mood disorders” OR “affective disorders” AND “adolescents.” This search will find all the articles about mood disorders or affective disorders in adolescents. The researchers can use NOT if they only want to study depression in children and want to ignore bipolar disorder from the search. An example search in this scenario would be “depression” NOT “bipolar disorder” AND “children.” This will help ignore studies related to bipolar disorder in children.

Truncation and wildcards are other tools to make search strategy more comprehensive and focused. While the researchers search a database for certain articles, they frequently face terminologies that have the same initial root of a word but different endings. An example would be "autism," "autistic," and "autism spectrum disorder." These words have a similar initial root derived from “autis” but they end differently in each case. The truncation symbol (*) retrieves articles that contain words beginning with “autis” plus any additional characters. Wildcards are used for words with the same meanings but different spellings due to various reasons. For the words with spelling variations of a single letter, wildcard symbols can be used. When the researcher inputs “M+N” in the search bar, this returns results containing both “man” or “men” as the wildcard accounts for the spelling variations between the letters M and N.

### Study selection

Study selection should be performed in a systematic manner, so reviewers deal with fewer errors and a lower risk of bias (online course, Li T, Dickersin K: Introduction to systematic review and meta-analysis. 2016. https://www.coursera.org/learn/systematic-review#). Study selection should involve two independent reviewers who select studies using inclusion and exclusion criteria. Any disagreements during this process should be resolved by discussion or by a third reviewer [10]. Specific study types can be selected depending on the research question. For example, questions on incidence and prevalence can be answered by surveys and cohort studies. Clinical trials can provide answers to questions related to therapy and screening. Queries regarding diagnostic accuracy can be answered by clinical trials and cross-sectional studies (online course, Li T, Dickersin K: Introduction to systematic review and meta-analysis. 2016. https://www.coursera.org/learn/systematic-review#). Prognosis and harm-related questions should use cohort studies and clinical trials, and etiology questions should use case-control and cohort studies (online course, Li T, Dickersin K: Introduction to systematic review and meta-analysis. 2016. https://www.coursera.org/learn/systematic-review#).

Data screening and data extractions are two of the major steps in conducting a systematic review [18]. Data screening involves searching for relevant articles in different databases using keywords. The next step of data screening is manuscript selection by reviewing each manuscript in the search results to compare that manuscript against the inclusion criteria [18]. The researchers should also review the references of the papers selected before selecting the final paper, which is the last step of data screening [18].

The next stage is extracting and appraising the data of the included articles [18]. A data extraction form should be used to help reduce the number of errors, and more than one person should record the data [17]. Data should be collected on specific points like population type, study authors, agency, study design, humanitarian crisis, target age groups, research strengths from the literature, setting, study country, type(s) of public health intervention, and health outcome(s) addressed by the public health intervention. All this information should then be put into an electronic database [18].

### Assessing bias

Bias is a systematic error (or deviation from the truth) in results or inferences. Biases can change the results of any study and lead to an underestimation or overestimation of the true intervention effect [19]. Biases can impact any aspect of a review, including selecting studies, collecting and extracting data, and making a conclusion. Biases can vary in magnitude; some are small, with negligible effect, but some are substantial to a degree where an apparent finding may be entirely due to bias [19]. There are different types of bias, including, but not limited to, selection, detection, attrition, reporting, and performance.

Selection bias occurs when a sample selected is not representative of the whole general population. If randomization of the sample is done correctly, then chances of selection bias can be minimized [20].

Detection bias refers to systematic differences between groups in how outcomes are determined. This type of bias is based on knowledge of the intervention provided and its outcome [19].

Attrition bias refers to systematic differences between groups in withdrawals from a study [19]. The data will be considered incomplete if some subjects are withdrawn or have irregular visits during data collection.

Reporting bias refers to systematic differences between reported and unreported findings, and it is commonly seen during article reviews. Reporting bias is based on reviewer judgment about the outcome of selected articles [20].

Performance bias develops due to the knowledge of the allocated interventions by participants and personnel during the study [20]. Using a double-blind study design helps prevent performance bias, where neither the experimenter nor the subjects know which group contains controls and which group contains the test article [14].

### Last step of systematic review: discussion

The discussion of a systematic review is where a summary of the available evidence for different outcomes is written and discussed [10]. The limitations of a systematic review are also discussed in detail. Finally, a conclusion is drawn after evaluating the results and considering limitations [10].

### Discussion of the current article

Systematic reviews with or without a meta-analysis are currently ranked to be the best available evidence in the hierarchy of evidence-based practice [21]. We have discussed the methodology of a systematic review. A systematic review is classified in the category of filtered information because it appraises the quality of the study and its application in the field of medicine [21]. However, there are some limitations of the systematic review, as we mentioned earlier in our article. A large randomized controlled trial may provide a better conclusion than a systematic review of many smaller trials due to their larger sample sizes [22], which help the researchers generalize their conclusions for a bigger population. Other important factors to consider include higher dropout rates in large studies, co-interventions, and heterogeneity among studies included in the review.

As we discussed the limitations of the systematic review and its effect on quality of evidence, there are several tools to rate the evidence, such as the Grading of Recommendations Assessment, Development and Evaluation (GRADE) system [22]. GRADE provides a structured approach to evaluating the risk of bias, serious inconsistency between studies, indirectness, imprecision of the results, and publication bias [22]. Another approach used to rate the quality of evidence is a measurement tool to assess systematic reviews (AMSTAR) [23]. It is also available in several languages [23].

## Conclusions

Despite its limitations, a systematic review can add to the knowledge of the scientific community especially when there are gaps in the existing knowledge. However, conducting a systematic review requires different steps that involve different tools and strategies. It can be difficult at times to access and utilize these resources. A researcher can understand and strategize a systematic review following the different steps outlined in this literature review. However, conducting a systematic review requires a thorough understanding of all the concepts and tools involved, which is an extensive endeavor to be summed up in one article.

The Cochrane Handbook for Systematic Reviews of Interventions and the Center for Reviews and Dissemination (CRD) provide excellent guidance through their insightful and detailed guidelines. We recommend consulting these resources for further guidance.

Given that our article is a narrative review of the scholarly literature, it contains the same limitations as noted for any narrative review. We hope that our review of the means and methods for conducting a systematic review will be helpful in providing basic knowledge to utilize the resources available to the scientific community.
